# Neuroglial P2Y_1_ receptor signalling differentially contributes to inflammatory neurodegeneration

**DOI:** 10.1186/s12974-026-03904-1

**Published:** 2026-06-13

**Authors:** Charlotte Schubert, Ricardo Lopes Fonseca, Alexandros Hadjilaou, Vanessa Vieira, Karoline Degenhardt, Anna Lena Seemann, Alice Hakimy, Jana K. Sonner, Peter Ludewig, Tim Magnus, Marion Schneider, Christa E. Müller, Daniela Hirnet, Manuel A. Friese

**Affiliations:** 1 Institute of Neuroimmunology and Multiple Sclerosis (INIMS), University Medical Center Hamburg-Eppendorf, Hamburg, Germany; 2 Protozoa Immunology, Bernhard-Nocht-Institute for Tropical Medicine (BNITM), Hamburg, Germany; 3 German Center for Infection Research (DZIF), Partner Site Hamburg-Lübeck-Borstel-Riems, Hamburg, Germany; 4 Department of Neurology, University Medical Center Hamburg-Eppendorf, Hamburg, Germany; 5 Pharmaceutical Institute, Pharmaceutical & Medicinal Chemistry, University of Bonn, Bonn, Germany; 6 Department of Animal Physiology, University of Hamburg, Hamburg, Germany

**Keywords:** EAE, Neuroinflammation, Neuron-glial crosstalk, Adenine nucleotides, Purine receptors

## Abstract

Extracellular adenosine triphosphate (ATP) and diphosphate (ADP) act as key signalling molecules in the central nervous system (CNS) and regulate neuroinflammatory responses through purinergic receptors. Although astrocytes and neurons undergo profound changes in signalling and metabolism during inflammation, the contribution of specific purinergic pathways to inflammation-induced neurodegeneration remains unclear. Here we show that the ADP/ATP-activated Gq-coupled receptor P2Y_1_ drives astrocyte-mediated neurotoxicity in experimental autoimmune encephalomyelitis (EAE), a model of multiple sclerosis (MS). Using plasma membrane-targeted luciferase reporter mice, we demonstrate that extracellular ATP levels are increased during acute EAE. This was accompanied by elevated astrocytic *P2ry1* expression, which is also observed in inflammatory MS lesions. In vivo, pharmacological inhibition or astrocyte-specific deletion of P2Y_1_ reduced disease severity, astrocytosis, and neuronal loss, whereas neuron-specific deletion exerted only modest effects. Mechanistically, astrocytic P2Y_1_ signalling promoted cytokine-induced ERK activation, inflammatory gene expression, and metabolic reprogramming in vitro. In contrast to supernatants from stimulated P2Y_1_-deficient astrocyte culture, supernatants derived from stimulated P2Y_1_-proficient astrocytes reduced neuronal viability, demonstrating neurotoxic effects mediated by astrocyte-derived factors. In contrast, neuronal P2Y_1_ signalling primarily contributed to oxidative stress and mitochondrial dysfunction. Together, these findings identify astrocytic P2Y_1_ as a key regulator of neuroinflammatory damage and a potential therapeutic target.

**Supplementary Information:**

The online version contains supplementary material available at 10.1186/s12974-026-03904-1.

## Background

Multiple sclerosis (MS) is a chronic autoimmune disease affecting more than 2.8 million people worldwide and represents the leading cause of neurological disability in young adults [1–3]. Treatments targeting acute inflammatory activity have become increasingly effective owing to the development of a broad range of immunomodulatory disease-modifying therapies [4]. Extensive research has shown the roles of infiltrating immune cells and activated microglia in driving neuronal dysfunction during relapses [5]. However, the mechanism underlying the progressive phase of MS, characterized by the gradual accumulation of neurological symptoms, remain poorly understood. Consequently, there is an unmet clinical need for therapeutic strategies that halt inflammation-induced neurodegeneration in MS [6, 7].

Astrocytes are the most abundant glial cells in the central nervous system (CNS) and perform critical homeostatic functions, including the regulation of neurotransmitters, ion gradients, and synaptic plasticity, as well as maintenance of the blood–brain barrier (BBB) integrity [8–10]. Although traditionally viewed as passive support cells, astrocytes are now recognized as dynamic regulators of CNS function engaging in bidirectional communication with neurons through ion channels, neurotransmitters, and metabolic pathways [11]. Increasing evidence implicates astrocytes as major contributors to neuroinflammatory diseases such as MS and its model, experimental autoimmune encephalomyelitis (EAE) [11–14]. During neuroinflammation, astrocytes undergo phenotypic remodelling, including hypertrophy, upregulation of glial fibrillary acidic protein (GFAP) and vimentin, and transcriptional shifts toward either pro-inflammatory or protective states [15]. Reactive astrocytes can amplify inflammation via the release of cytokines such as interleukin-1 β (IL-1β) and tumor necrosis factor-α (TNF-α), chemokines (CCL2, CXCL10), and reactive oxygen species (ROS), thereby promoting immune cell infiltration and neurotoxicity [16]. Conversely, they can exert neuroprotective functions by secreting anti-inflammatory mediators and trophic factors, supporting neuronal survival, synaptic remodelling, and metabolic coupling [17]. This remarkable functional plasticity enables astrocytes to dynamically balance neuroprotective and neurotoxic responses following CNS injury [18].

The purinergic signalling system, comprising extracellular nucleotides, receptors and metabolizing enzymes, is a fundamental and evolutionary conserved regulator of neuroinflammation [19, 20]. During MS and related neuroinflammatory conditions, elevated levels of danger-associated molecular patterns (DAMPs), including adenine nucleotides such as adenosine triphosphate (ATP) and adenosine diphosphate (ADP) [21], are released from damaged neurons, glial cells, and infiltrating immune cells [22, 23]. These purines activate P2X and P2Y receptors, triggering inflammatory and metabolic responses [24–26]. While in MS previous studies focused on the roles of P2X receptors and purinergic signalling in immune cells, the contribution of metabotropic P2Y receptors in CNS-resident cells remains less explored [27, 28]. Among these, the P2Y_1_ receptor, a key mediator of ATP/ADP signalling, has emerged as a candidate regulator of neuroinflammatory processes [29, 30].

Given the critical role of purinergic signalling in CNS inflammation, we investigated the contribution of ADP/ATP and their metabotropic receptor P2Y_1_ in EAE. We identified astrocytic P2Y_1_ as a critical mediator of inflammation-induced neurodegeneration both in vitro and in vivo. Activation of astrocytic P2Y_1_ enhances astrocyte reactivity, promoting metabolic reprogramming, cytokine release, and glutamate secretion, ultimately compromising neuronal survival.

## Methods

### Mice

All mice (C57BL/6J WT; The Jackson Laboratory) the pmeLUC mouse line expressing a bioluminescent ATP probe on the cell surface [31, 32], and the cell-type specific *P2ry1* knockout mouse lines *P2ry1*^*flx/flx*^*;GLAST-Cre*^*ERT2*^ [33, 34] (referred to as *P2ry1*-KO^astro^) and *P2ry1*^*flx/flx*^*;SNAP25-Cre* [35] (referred to as *P2ry1*-KO^neuro^) were maintained under specific pathogen-free conditions in the central animal facility of the University Medical Center Hamburg-Eppendorf (UKE). Littermate controls were Cre-negative mice carrying the homozygous floxed *P2ry1* allele (*P2ry1*^flx/flx^). We used adult mice (9–18 weeks old) from both sexes; mice were sex- and age-matched in all experiments. As we did not observe sex-specific differences in the experiments, data from males and females were pooled.

### Tamoxifen-induced gene recombination

To induce the astrocyte-specific knockout *P2ry1*-KO^astro^ mice were injected intraperitoneally with tamoxifen (TAM, Thermo Scientific) in Mygliol® 812 (Azelis Pharma, 100 mg/kg body weight, 10 mg/ml TAM in Miglyol® 812) once daily for five consecutive days. Experiments were performed 4–5 weeks after injection to receive optimized reduction of *P2ry1* expression [34].

### EAE induction

For EAE induction, 9–13-week-old C57BL/6J mice were anesthetised with isoflurane 1–2% v/v oxygen and immunised subcutaneously with 200 µg myelin oligodendrocyte glycoprotein 35–55 (MOG_35–55_) peptide (peptides and elephants, Cat. # EP02030_1) emulsified in Complete Freund’s adjuvant (CFA; BD Difco, Cat. #DF0638-60-7) containing 4 mg mL^–1^ *Mycobacterium tuberculosis* (BD Difco, Cat. #DF3114-33-8). Pertussis toxin (200 ng; Merck Millipore, Cat. #516560) was injected intraperitoneally in 100 µL PBS on day of immunisation and again two days later. Body weight and clinical signs were monitored daily from day 7 to day 30. Mice were scored for clinical signs by the following system: 0: no clinical deficits; 1: tail weakness; 2: hind limb paresis; 3: partial hind limb paralysis; 3.5: full hind limb paralysis; 4: full hind and forelimb paresis. Animals that reached a score of 4, or 3.5 for more than 7 days, or lost ≥ 25% of their initial body weight were euthanised according to local Animal Welfare Act regulations.

### Extracellular ATP measurement

Luminescence emission in pmeLuc mice was measured at the acute phase of EAE and during healthy control condition with a whole-body luminometer for small animals (IVIS Lumina, Perkin Elmer). Mice anesthetized with 2% isoflurane were injected intraperitoneally with 150 mg kg^–1^ D-luciferin Firefly (Biosynth) and measured after a 15-min interval to allow biodistribution. Luminescence signals were acquired from the dorsal side. The animals were shaved on the dorsal side prior to the experiment. Photon emission was quantified using the Living Image® software (Perkin Elmer) and expressed as photons/seconds/cm2/steradian (abbreviated as p/s/cm2/sr).

### Single-cell RNA sequencing analysis

First, we re-analyzed a publicly available snRNA-seq dataset of mouse spinal cord (GSE281176) [36]. Raw 10 × Genomics H5 files from eight vehicle-treated samples were processed using Seurat v5. Nuclei with 200–6,000 detected genes and < 10% mitochondrial reads were retained. Samples were normalized using SCTransform v2 (glmGamPoi, regressing percent.mt, 3,000 variable features) and integrated via reciprocal PCA (30 dimensions, largest sample as reference). UMAP embedding and shared nearest-neighbour clustering (resolution 0.5) yielded 19 clusters. Cell types were assigned by scoring clusters against curated canonical marker gene panels and by reference-based classification using SingleR (ImmGen and MouseRNAseq references from celldex). For downstream *P2ry1* analyses, only naive samples were used. Astrocytes were selected from the existing scRNA-seq dataset based on the cluster annotations used. Five astrocyte clusters were visualised within the existing UMAP embedding. *P2ry1*-positive cells were defined as cells with detectable *P2ry1* expression (P2ry1 > 0) and highlighted within the astrocyte population. The frequency of *P2ry1*-positive cells was quantified separately for each astrocyte cluster. Second, we re-analysed a published snRNA-seq dataset of post-mortem human white matter (GSE180759) comprising 66,432 nuclei from five progressive MS brains and three non-neurological controls [37]. The expression matrix and author-provided annotations were downloaded from GEO. Data were log-normalized, and PCA (30 components) followed by UMAP were computed for visualization. Cell type and pathology labels were taken from the published metadata without re-annotation. Third, spatial gene expression was analysed using a published 10 × Visium dataset (GSE279181) comprising sections from three MS patients and two controls [38]. Pre-processed H5AD files with niche annotations (lesion rim, lesion core, periplaque white matter, grey matter, vasculature/immune) were loaded using AnnData. Per-spot counts were normalized to log1p (counts per 10,000). To assess astrocyte-specific *P2ry1* enrichment, expression was normalized by the deconvolution-derived astrocyte proportion per spot. Statistical comparisons between niches used two-sided Mann–Whitney U-tests with Benjamini–Hochberg correction.

### RNAscope in situ hybridization

RNAscope in situ hybridization was performed on spinal cord tissue using the RNAscope™ 2.5 HD Assay – RED (Advanced Cell Diagnostics, Cat. #322350) according to the manufacturer’s instructions. Animals were transcardially perfused with phosphate-buffered saline (PBS) followed by 4% paraformaldehyde (PFA). Spinal cords were post-fixed in 4% PFA for 24 h at 4 °C and cryoprotected in a sucrose gradient (10% for 18 h, 20% for 6 h and 30% for 4 days). Tissue was cryosectioned at 12 µm thickness without embedding in OCT compound. Sections were processed for RNAscope using a probe targeting *P2ry1* (Advanced Cell Diagnostics, Cat. #4060641). After completion of the RNAscope assay and chromogenic detection, sections were subjected to immunofluorescence staining with antibodies against GFAP (anti-GFAP; 1:500, Sigma Aldrich, Cat. #AB5541) and HuC/HuD (anti-HuC/HuD; 1:500, Invitrogen, Cat. #A21271). Images were acquired using a confocal microscope under identical acquisition settings for all experimental conditions.

### Astrocyte isolation and flow cytometric sorting

Astrocytes were isolated from female C57BL/6J mice (10 weeks old) that were either healthy or EAE induced. CNS tissues (brain and spinal cord) were collected at disease onset (d10–11 p.i.), during acute (d14–15 p.i.), and during chronic EAE (d29–30 p.i.) and processed immediately. In addition, astrocytes were isolated from female and male *P2ry1*-KO^astro^ mice (16–18 weeks old) following tamoxifen-induced gene recombination. Tissues were enzymatically dissociated using activated papain (50 U mL^–1^; Worthington) and DNase I (250 U mL^–1^; Sigma) in Ca^2^⁺ and Mg^2^⁺-free HBSS for 30 min at 37 °C with gentle agitation using a gentleMACS™ Dissociator (Miltenyi Biotec). Digestion was terminated with ice-cold HBSS containing 2 mM EDTA. Cell suspensions were purified by low-endotoxin PercollPLUS density centrifugation (350 × g, 5 min, 4 °C) and resuspended in HBSS supplemented with 0.1–0.5% BSA and 2 mM EDTA. Following Fc receptor blocking and live/dead staining, cells were labelled with fluorochrome-conjugated antibodies. Astrocytes (ACSA-2⁺CD45⁻O4⁻CD49a⁻), microglia (CD45^dim^CD11b^hi^Ly6C^lo^), oligodendrocytes (O4⁺CD45⁻), and T cells (CD45⁺⁺TCRβ⁺; C57BL/6J mice) or endothelial cells (CD49a⁺CD45⁻; *P2ry1*-KO^astro^ mice) were sorted using a BD FACSAria™ (100 µm nozzle, 4 °C). Up to 2 × 10^5^ cells per population were collected into HBSS containing 0.5% BSA and immediately lysed in RLT buffer for RNA extraction (RNeasy Mini Kit, Qiagen). Post-sort purity and viability were confirmed by reanalysis.

### Quantitative real-time PCR

Isolated RNA was reversed-transcribed into cDNA with the RevertAid H Minus First Strand cDNA Synthesis Kit (Thermo Fisher Scientific) according to the manufacturer’s instructions. Gene expression was analysed by real-time PCR performed on an ABI Prism 7900 HT Fast Real-Time PCR System (Applied Biosystems) using TaqMan Gene Expression Assays (Thermo Fisher Scientific) of the genes listed in Supplementary Table 2. Relative transcript expression was calculated as 2^–ΔCt^ method with *Tbp* as the endogenous control.

### Intranasal treatment

Beginning on day seven post EAE induction and continuing daily until day 30, EAE mice and corresponding control groups received intranasal administration of the P2Y_1_ receptor antagonist MRS 2179 (Tocris, Cat. #0900) or PBS. The MRS 2179 dose was 10 mg kg^–1^ body weight, calculated based on the baseline weight prior to treatment. The total administration volume was 20 µL per mouse, delivered as two 5 µL aliquots per nostril. During administration, mice were positioned in a ventroflexed head posture using a custom apparatus to ensure optimal compound distribution within the brain. Intranasal application was performed under light anaesthesia (1–2% isoflurane). Mice were monitored daily throughout the treatment period and showed no signs of toxicity.

### Mouse tissue preparation and immunohistochemistry

For immunohistochemistry, mice were anesthetized via intraperitoneal injection with 100 µL per 10 g body weight of a solution containing 10 mg mL^–1^ esketamine hydrochloride (Pfizer) and 1.6 mg mL^–1^ xylazine hydrochloride (Bayer) in water. Following anaesthesia, animals were perfused transcardially with PBS, followed by 4% paraformaldehyde (PFA) for fixation.

### Liquid chromatography coupled to mass spectrometry (LC–MS)

Mouse brains were dissected. Brains were added to 1 ml of acetonitrile each. After treatment for 5 min in a TissueLyser (a bead mill homogenizer; Qiagen, Hilden, Germany) at 50 Hz and subsequent homogenization in an ultrasonic bath followed by vortexing (2 min each), the samples were centrifuged at 20,000 g for 15 min. An aliquot of the supernatant (0.2 ml) was subsequently transferred to an HPLC vial. The mass spectra were recorded on a QTrap 6500 + (Sciex, Darmstadt, Germany) with an ESI-source coupled with an HPLC 1290 Infinity (Agilent, Waldbronn, Germany) using a Synergi Fusion RP HPLC column (Phenomenex, Aschaffenburg, Germany). The column temperature was 30 °C. Multiple reaction monitoring (MRM) was used, detecting a positive MRM from 425.7 u to 150.0 u. The HPLC gradient started after 3 min with 100% water containing 2 mM ammonium acetate. Within 2 min, 100% methanol containing 2 mM ammonium acetate was reached, followed by flushing the column with methanol containing 2 mM ammonium acetate for 2.5 min and subsequent equilibration for 2.5 min with 100% water containing 2 mM ammonium acetate. Sample solution (5 µL) was injected to a flow rate of 0.6 mL/min. A calibration curve for MRS2179 was recorded, and the recovery rate was determined. The detection limit was around 0.1 nM. The recovery rate was ca. 40%. The determined concentration was 1.91 ± 0.52 nM. Concentration was calculated per gram brain tissue in ng/g.

### Histopathology

For histopathological analysis, brain tissue was fixed in 4% PFA as described above. After four hours of fixation, tissues were transferred to PBS, decalcified, dehydrated, embedded in paraffin, and processed according to the standard procedures of the UKE Mouse Pathology Facility. For tissue orientation, hematoxylin staining (blue) was performed, followed by immunostaining with, anti-NeuN (rabbit IgG, Millipore, Cat. #MAB377), anti-GFAP (rabbit IgG, Merck, Cat. #MAB3402), anti-Iba1 (rabbit IgG, Fujifilm Wako, Cat. #019–19741) and anti-CD3 (rabbit IgG, Abcam, Cat. #ab16669). Primary antibodies were visualized using the avidin–biotin complex method with 3,3’-diaminobenzidine (DAB, brown stain). Slides were imaged using a NanoZoomer 2.0-RS digital slide scanner. NeuN⁺ neurons were quantified specifically within the ventral horn of the spinal cord, whereas CD3⁺ T cells, Iba1⁺ microglia/macrophages, and GFAP immunoreactivity were analysed across the entire spinal cord section. CD3⁺, NeuN⁺, Iba1^+^ cells were quantified by cell counting, whereas GFAP and Iba1 immunoreactivity were assessed based on signal intensity and area using QuPath (https://qupath.github.io/). GFAP immunoreactivity was quantified on brightfield images following H-DAB colour deconvolution to separate hematoxylin and DAB signals. Quantification was performed on the DAB channel, representing GFAP staining intensity. To distinguish reactive from baseline GFAP expression, an intensity threshold was applied to the DAB optical density signal. The threshold was applied uniformly across all images to exclude low-intensity baseline staining. All images were acquired and analysed using identical settings to ensure comparability between groups. All quantifications were performed in a blinded manner.

### Primary astrocytic culture

Primary glial cells were prepared from the cortices of postnatal day 0 (P0) mice. Cortical tissue was dissected and mechanically dissociated in Hank’s Balanced Salt Solution (HBSS) at room temperature (RT), followed by enzymatic digestion with 0.25% trypsin (Thermo Fisher Scientific, Cat. #25200-072) supplemented with DNase I (Roche, Cat. #11284932001) for 15 min at 37 °C. After incubation, the samples were centrifuged for 5 min at 300 × g to pellet the tissue, and the pellet was resuspended in astrocyte culture medium (ACM; Dulbecco’s Modified Eagle’s Medium (DMEM; Gibco, Cat. #11965092) supplemented with 10% foetal calf serum (FCS; Sigma-Aldrich, Cat. #F7524) and 1% penicillin–streptomycin (Gibco, Cat. #15140122). The degree of dissociation was assessed by counting single cells using a haemocytometer. Typically, cortices from four mouse pups yielded 10–15 × 10⁶ single cells. The dissociated cells were plated into T75 tissue culture flasks and maintained in ACM, which was replaced every two days with fresh pre-warmed medium. Before the first passage, cells were cultured for 14 days. After 7–10 days in vitro, microglia were removed by differential adhesion. Culture flasks were gently shaken manually for 10–15 min to detach weakly adherent microglia. The medium containing detached cells was carefully aspirated and replaced with fresh pre-warmed ACM. This procedure was repeated once after 24 h. Seven to ten days after the first split, astrocytes were plated at the desired density 24–48 h before experiments.

### rAAV transduction of primary astrocytes

Primary astrocytic cultures (PACs) were generated from P2ry1^flx/flx^ mice as described above. To induce gene deletion, astrocytes were transduced in vitro with recombinant adeno-associated virus (rAAV) encoding Cre recombinase under the control of a CMV promoter, whereas control cultures received a corresponding Cre-negative rAAV. Transduction was performed 14 days after astrocyte plating, and cells were subsequently maintained under standard culture conditions for an additional 7–10 days to allow efficient recombination before use in mechanistic experiments. Culture medium was refreshed every 2–3 days.

### Primary neuronal cortical culture

Primary cortical cultures were prepared from E16.5 mouse embryos. Cortical tissue was incubated in 0.05% trypsin–EDTA (Gibco, Cat. #25300054) for 6 min at 37 °C, and digestion was stopped with DMEM-F12 containing 10% FCS. The tissue was then dissociated in HBSS and centrifuged for 2 min at 500 × g. The pellet was resuspended in Primary Growth Medium (PGM), and cells were plated at 1 × 10^5^ per cm^2^ on poly-d-lysine-coated plates. We maintained cultures in PNGM (Primary Neuron Growth Medium BulletKit, Lonza, Cat. #CC-4461) at 37 °C, 5% CO_2_ and 98% relative humidity. To inhibit glial proliferation, we added cytarabine (AraC, 1 µM; Sigma, Cat. #C6645), and cultures were maintained for 14–18 days in vitro (DIV).

### Astrocyte-neuronal co-culture

Primary neurons and astrocytes were prepared as described above. Neurons were seeded on 12-mm glass coverslips placed in the lower compartment of 24-well plates. Confluent astrocyte cultures were rinsed twice with PBS, then incubated with 5 mL TrypLE Express (Gibco, Cat. #12604054) at 37 °C in a CO₂ incubator. Detachment of astrocytes was monitored every 5 min and, if necessary, aided by gently tapping the flask. Once detached, 5 mL ACM was added to stop the enzymatic reaction. Cells were collected by centrifugation at 180 × g for 5 min, resuspended in 10 mL fresh astrocyte plating medium, and counted using Trypan blue exclusion in a Neubauer counting chamber. Astrocytes were plated at a density of 6 × 10^4^ cells per cm^2^ on cell culture inserts (Greiner Cell Coat, Cat. #10482885), initially maintained in a separate 24-well plate. After cell attachment, the inserts were transferred into the wells containing neurons in the lower compartment. Co-cultures were maintained for at least 72 h under standard conditions (37 °C, 5% CO₂) before experiments were performed.

### Immunofluorescence

Primary mouse neurons and astrocytes were cultured on 12-mm glass coverslips. Cells were fixed with 4% PFA and incubated in 10% normal donkey serum (NDS) containing 0.1% Triton X-100 for permeabilization and blocking. Neuronal morphology was visualized with an antibody against microtubule-associated protein 2 (MAP2; 1:500, Merck, Cat. #AB5543). Astrocytes were identified using an antibody against glial fibrillary acidic protein (GFAP; 1:1,000, Sigma-Aldrich, Cat. #G3893). For astrocytic signalling analyses, antibodies against phosphorylated ERK (pERK; 1:500, Invitrogen, Cat. #36-8800), phosphorylated AKT (pAKT; 1:500, Cell Signaling Technology, Cat. #9271), and phosphorylated p38 MAPK (p-p38; 1:500, Cell Signaling Technology, Cat. #9211) were used. Alexa Fluor–conjugated secondary antibodies (Thermo Fisher Scientific) were applied for fluorescence detection. A complete list of antibodies used in this study can be found in Supplementary Table 1. Images were acquired using a Zeiss LSM 700 laser scanning confocal microscope.

### Cell viability assay (DAPI)

Primary murine cortical neurons (DIV14) were used for all experiments. Cells were stimulated as described in the corresponding experimental sections. After 4–6 h of stimulation, 5 µM DAPI (Invitrogen) was added to the culture medium for 10 min, after which cells were fixed and immunostained for the neuronal marker MAP2 (ABCAM, Cat. #AB5392) and propidium iodide (PI; 1:1,000; BioLegend, Cat. #421301). Neuronal nuclei served as region of interest (ROI) for quantifying DAPI uptake by mean fluorescence intensity (MFI), which was used as a measure of increased membrane permeability. Propidium iodide (PI) was used post-fixation as a nuclear counterstain to visualise total cell nuclei independently of membrane integrity. At least three technical replicates were included per condition. In experiments in which DAPI was applied after fixation, it was used exclusively as a nuclear counterstain and not as a measure of cell viability. The timing and purpose of DAPI application are specified in the respective figure legends.

### Lactate and glutamate secretion assay

Primary astrocytes were prepared as described above. Prior to stimulation, the culture medium was replaced with fresh ACM. Cells were then treated with TNF-α (10 ng mL^–1^; Pan Biotech, Cat. #CB-1212011M) and IL-1β (10 ng mL^–1^; Gibco, Cat. #211-11B-10UG) for 6–8 h, as indicated. After stimulation, supernatants were collected, diluted, and stored according to the manufacturer’s instructions for the Lactate- and Glutamate-assay (Promega, Cat. #J5021, #J7021). A standard curve was generated for metabolite quantification. Luminescence was measured using a Spark 10 M multimode microplate reader (Tecan), and metabolite secretion was quantified according to the manufacturer’s protocol. At least 3 technical replicates per condition were used.

### Neuro2a cell-line

Neuro-2a (N2A) mouse neuroblastoma cells were maintained in Dulbecco’s Modified Eagle Medium (DMEM, high glucose) supplemented with 10% foetal bovine serum (FBS), 1% penicillin–streptomycin, and 2 mM L-glutamine at 37 °C in a humidified incubator with 5% CO₂. Culture medium was routinely replaced every 2–3 days. Cells were passaged at 70–80% confluency using 0.05% trypsin–EDTA and reseeded at appropriate densities depending on the experimental setup. For passaging, cells were washed once with phosphate-buffered saline (PBS), detached by brief trypsinization, and the reaction was stopped by addition of complete culture medium. Cells were subsequently collected by centrifugation, resuspended in fresh medium, and replated.

For experimental procedures, cells were seeded onto tissue culture-treated plates and allowed to adhere and recover for at least 18–24 h prior to treatment. Seeding densities were adjusted according to the respective downstream applications, including microscopy-based assays or biochemical analyses. Cell cultures were routinely monitored for morphology and growth characteristics and were periodically tested for mycoplasma contamination. Only low-passage cells were used to ensure experimental reproducibility.

### mHippo-E14 mouse hippocampal neuronal cells

mHippo-E14 mouse hippocampal neuronal cells were maintained in Dulbecco’s Modified Eagle Medium (DMEM, high glucose) supplemented with 10% foetal bovine serum (FBS), 1% penicillin–streptomycin, and 2 mM L-glutamine at 37 °C in a humidified incubator with 5% CO₂. Culture medium was routinely replaced every 2–3 days. Cells were passaged at approximately 70–80% confluency using 0.05% trypsin–EDTA and reseeded at appropriate densities depending on the experimental requirements. For passaging, cells were washed once with phosphate-buffered saline (PBS), detached by brief trypsinization, and the reaction was stopped by addition of complete culture medium. Cells were then collected by centrifugation, resuspended in fresh medium, and replated.

For experimental procedures, cells were seeded onto tissue culture-treated plates and allowed to adhere and recover for at least 18–24 h prior to treatment. Seeding densities were adjusted according to the respective downstream applications. Cell cultures were routinely monitored for morphology and growth characteristics and were regularly tested for mycoplasma contamination. Only low-passage cells were used to ensure reproducibility and consistency of experimental results.

### Neuro2a neuronal cell line viability assay

Ten thousand Neuro2a neuronal cells (ATCC, CCL-131) were seeded per well in 96-well plates and preactivated with 100 ng mL^–1^ mouse IFN-γ (R&D Systems, Cat. #485-MI-100) for 18–24 h. After activation, the medium was removed and cells were washed with 1 × PBS before adding 200 µL ACM to each well. After 18–24 h incubation, cell viability was assessed using the LDH-Glo™ Cytotoxicity Assay (Promega, Cat. #2380) according to the manufacturer’s instructions. Luminescence was measured using a microplate reader. Cell viability was calculated by normalizing sample luminescence to the mean spontaneous LDH release (medium control) and maximal LDH release (lysis control) using the formula: Viability = 1 − (RLU_sample − RLU_low)/(RLU_high − RLU_low). Maximum LDH release was determined by lysing cells with 1% Triton X-100 (final concentration) for 10–15 min. At least 3 technical replicates per condition were used.

### Quantification and statistical analysis

**Fig. 1:**
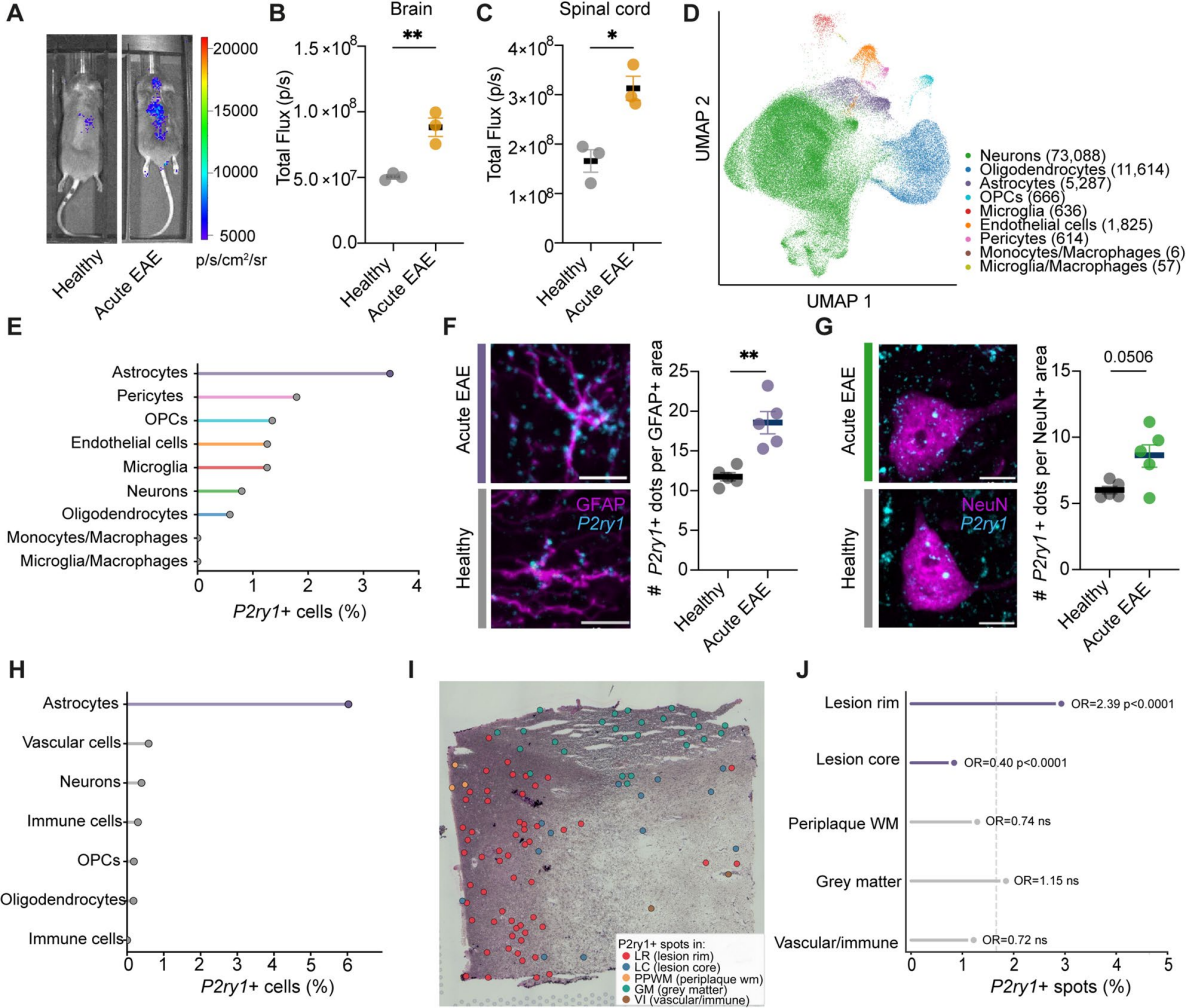
Elevated extracellular ATP and astrocytic *P2ry1* upregulation during CNS inflammation. **A** In vivo bioluminescence imaging of *pmeLuc* reporter mice used to quantify ATP levels in CNS compartments. Images show healthy control mice and acute phase EAE mice at day 15 post-immunisation (p.i.). **B** Quantification of total photon emission per second in cerebral compartment of *pmeLuc* reporter mice, comparing healthy controls and acute EAE mice at day 15 p.i. (*n* = 3 per group). **C** Quantification of total photon emission per second in the spinal compartment in *pmeLuc* reporter mice, comparing healthy controls and acute EAE mice at day 15 p.i. (*n* = 3 per group). **D** Analysis of a single cell sequencing data (GSE281176, *n* = 4 animals [36]) comprising 93,793 nuclei from healthy C57BL/6J mouse spinal cord. UMAP of cell type clusters. Cell types were annotated based on canonical marker gene expression and SingleR reference-based classification. **E** Percentage of P*2ry1*-expressing cells across all cell clusters in the healthy mouse spinal cord. **F** Representative images and quantification of *P2ry1* mRNA expression measured by RNAscope in astrocytes of the spinal cord. *P2ry1* puncta were normalized to the GFAP-positive area to account for regional differences in astrocyte density (*n* = 5 per group). **G** Representative images and quantification of *P2ry1* mRNA expression measured by RNAscope in neurons of the spinal cord. *P2ry1* puncta were normalized to the NeuN-positive area (*n* = 5 per group). Scale bar in (**F**) and (**G**) = 10 µm. **H** Percentage of *P2RY1*-expressing nuclei per cell type in human non-inflammatory post-mortem white matter (GSE180759; 66,432 nuclei, 12 samples [37]). Cell type annotations from Absinta et al. **I** Visualisation of *P2ry1* + spots in MS brain tissue in spatial transcriptomics comprising a chronic active lesion with a lesion rim (LR), lesion core (LC), periplaque white matter (PPWM), grey matter (GM), vasculature and immune cells (VI), (GSE279181 [38]) and **J** Quantification of data set visualised in (**I**) by lollipop plot of percentage of *P2ry1* + spots. Data are presented as mean ± SEM (**B**, **C**, **F** and **G**). In **B**, **C**, **F** and **G** unpaired t-tests were performed. In **E** and **H** proportions of *P2ry1*⁺ cells across cell types were calculated from single-cell datasets. In **J** enrichment of *P2ry1*⁺ spots across lesion regions was assessed using Fisher’s exact test and reported as odds ratios (OR); *P < 0.05, **P < 0.01

Image data were analysed using Fiji software (NIH). Experimental data were processed and analysed using GraphPad Prism 10. Unless otherwise stated, data are presented as mean ± standard error of the mean (SEM). Differences between two experimental groups were assessed using unpaired, two-tailed Student’s *t*-tests. For multiple comparisons, the false discovery rate (FDR) correction was applied. For EAE experiments, clinical scores were analysed using the Mann–Whitney U-test on the area under the curve (AUC) for each animal. Correlation analyses were performed using Pearson correlation. Significant differences were defined as follows: *P < 0.05, **P < 0.01, ***P < 0.001. snRNA-seq analyses used R v4.4.1 with Seurat v5, SingleR, celldex, and glmGamPoi. analyses of spatial transcriptomics used Python 3 with AnnData and SciPy. Figures for Fig. 1D, E, H-J were generated with ggplot2/patchwork (R) and matplotlib/seaborn (Python).

## Results

### ATP release and astrocytic *P2ry1* induction during CNS inflammation

To determine whether extracellular ATP levels are altered under neuroinflammatory conditions, we measured ATP dynamics using plasma membrane-targeted luciferase *(pmeLUC)* reporter mice [32]. During the acute phase of EAE, luminescence signals were significantly increased in the mouse brain and spinal cord (Fig. 1A-C), indicating elevated extracellular ATP. ATP and its metabolite signal through ionotropic and metabotropic P2 receptors. Because the P2Y_1_ receptor has not been previously evaluated during neuroinflammation, we analysed publicly available single cell RNA-sequencing data from spinal cord tissue [36] to identify *P2ry1*-expressing cells (Fig. 1D, E). Overall, *P2ry1* expression was most prominent in astrocytes, with subtle differences observed among astrocyte subclusters (Supplementary Fig. S1A). Using RNAscope, we validated that *P2ry1* expression was significantly upregulated in GFAP-positive astrocytes in the spinal cord during the acute phase of EAE (Fig. 1F). Neuronal *P2ry1* expression measured by RT-PCR showed a similar trend but did not reach significance (Fig. 1G). *P2ry1* mRNA-expression of isolated astrocytes by flow cytometry sorting revealed comparable results during the acute EAE; however, *P2ry1* expression was significantly reduced during the chronic phase (Supplementary Fig. S1B-D). In human post mortem brain tissue *P2ry1* expression quantified by RNAscope was highest in astrocytes (Fig. 1H) [37]. Re-analysis of a spatial transcriptomic data set of people with relapsing MS revealed an accumulation of *P2ry1* in the rim of the inflammatory lesions emphasizing the relevance of *P2ry1* in the diseased condition (Fig. 1I, J) [38].

Together, these data demonstrate that extracellular ATP is elevated during acute neuroinflammation and that P2ry1 is selectively upregulated in astrocytes in both murine EAE and human MS, supporting a potential role for astrocytic P2Y_1_ signalling in inflammatory CNS pathology.

### Astrocyte-specific deletion of P2Y_1_ attenuates EAE severity

**Fig. 2:**
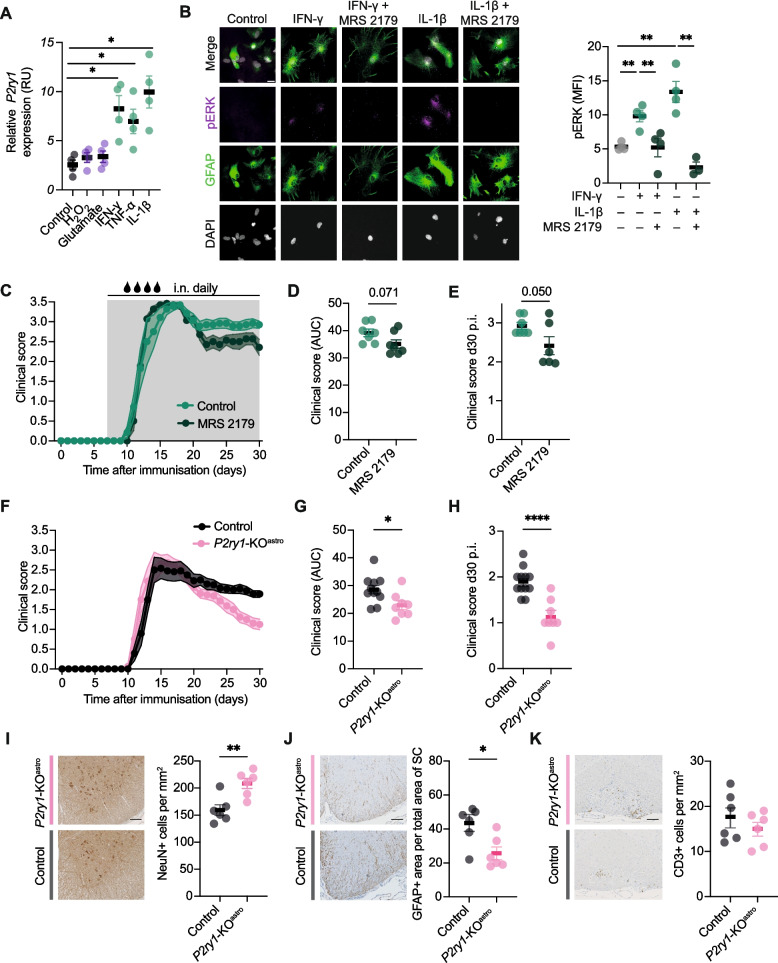
Astrocyte-specific deletion of P2Y_1_ attenuates EAE severity. **A** *P2ry1* mRNA expression (relative unit [RU]) in primary astrocytic cultures (PAC) under control condition or after treatment with glutamate (50 µM), H_2_O_2_ (100 µM), IFN-γ (10 ng mL^–1^), TNF- α (10 ng mL^–1^) or IL-1β (1 ng mL^–1^) for 24 h (*n* = 4). **B** Representative images and quantification of phospho-ERK1/2 (pERK1/2) mean fluorescence intensity (MFI) in primary astrocytes following 24 h stimulation with IFN-γ or IL-1β. Where indicated, cells were pretreated for 1 h with the P2Y_1_ antagonist MRS 2179 *(n* = 3–4*).* Scale bar = 20 µm. **C** Clinical EAE score of C57BL/6J mice treated daily intranasally (i.n.) from day 7 to day 30 post-immunisation with PBS (control) or MRS 2179 (10 mg kg^–1^, i.n.; *n* = 7 per group). The treatment period is indicated in grey. **D**-**E** Clinical EAE score (**D**) measured by area under the curve (AUC) during peak and chronic disease stage (15–30 days p.i.) and **E** at day 30 p.i., treatment was done daily intranasally (i.n.) from day 7 to day 30 post-immunisation with PBS (control) or MRS 2179 in C57BL/6J mice (control, *n* = 7; treatment-group, *n* = 6). **F** Clinical EAE score of *P2ry1* conditional knockout mice (*P2ry1-KO*^*astro*^, *n* = 9) and littermate controls (control, *n* = 13). **G**-**H** Clinical EAE score (**G**) measured by area under the curve (AUC) during peak and chronic disease stage (15–30 days p.i.) and **H** at day 30 p.i. (control, *n* = 12; *P2ry1-KO*^*astro*^, *n* = 8). **I**-**K** Representative histopathological images and quantification of (**I**) neuronal loss (NeuN), **J** astrocytic activation (GFAP) and **K** T-cell infiltration (CD3) during the chronic phase, 30 days after immunisation (all groups, *n* = 6). NeuN-positive cells were quantified as cells per mm^2^ in the ventral horn of the spinal cord (SC), whereas GFAP-positive area per total area and number of CD3-positive cells were quantified across the entire spinal cord section. Scale bar in (**I**–**K**) = 100 µm. In **B** DAPI was applied after fixation and is shown exclusively as a nuclear counterstain. Data are presented as mean ± SEM (**A**, **B**, **D**, **F**–**I**). In **A** one-way ANOVA with FDR correction was performed. In **B** data were analysed using two-way ANOVA with FDR correction. In **C** and **F** clinical scores were analysed using two-way repeated-measures ANOVA with FDR correction. For **D** and **F**–**I** two-sided Mann–Whitney tests were performed.; *P < 0.05, **P < 0.01, ***P < 0.001, ****P < 0.0001

Based on these results, we next focused on the role of astrocytic P2Y_1_ during neuroinflammation. In primary astrocytic cultures (PACs), *P2ry1* expression measured by RT-PCR was higher compared with primary neuronal cultures (PNCs) and the neuronal mouse line mHippo-E14 (Supplementary Fig. S2A). Following stimulation with interferon-γ (IFN-γ), TNF-α or IL-1β for 24 h to simulate an inflammatory milieu, *P2ry1* upregulation was evident, while glutamate or H_2_O_2_ exposure showed no differences (Fig. 2A). Immunohistochemistry revealed activation of phosphorylated extracellular signal-regulated kinase 1/2 (pERK1/2), but not p38 or pAkt, after pharmacological activation of P2Y_1_ by the agonist MRS 2365 (Supplementary Fig. S2B-D). ERK phosphorylation was further enhanced upon stimulation with the non-selective P2Y_1_/P2Y_12_/P2Y_13_ receptor agonist 2-methylthioadenosine diphosphate, indicating that engagement of additional P2Y receptors amplifies the pERK1/2 signal (2-MeSADP; Supplementary Fig. S2B). Furthermore, pERK1/2 levels were increased after IL-1β and IFN-γ treatment for 24 h (Fig. 2B), whereas co-treatment with the selective P2Y_1_ antagonist MRS 2179 significantly attenuated this effect (Fig. 2B).

To evaluate the therapeutic potential of P2Y_1_ inhibition in vivo, we administered the P2Y_1_-selective antagonist MRS 2179 intranasally on a daily basis to EAE mice starting at day seven post-induction. This treatment showed a trend toward reduced EAE severity during the chronic phase (Fig. 2C-E; Supplementary Fig. S2E), although no histological evidence of neuronal preservation was observed (Supplementary Fig. S2F). Consistent with this, analysis of the area under the curve (AUC) revealed only a modest reduction in overall disease burden (Fig. 2D). Intranasal administration was chosen to enhance CNS penetration; however, drug concentrations measured in brain tissue by high-performance liquid chromatography coupled to tandem mass spectrometry remained relatively low (Supplementary Fig. S2G), suggesting insufficient delivery of the P2Y_1_ antagonist to the CNS parenchyma. Together, these findings indicate that although pharmacological inhibition of P2Y_1_ showed a trend toward clinical benefit, the limited efficacy observed in this setting likely reflects suboptimal CNS target engagement.

To probe cell type-specific mechanisms, we generated and validated a conditional knockout model (Supplementary Fig. S2H). Astrocyte-specific deletion of *P2ry1* (*P2ry1-KO*^*astro*^) resulted in significantly lower EAE severity scores during the chronic phase compared to wildtype littermate controls (Fig. 2F-H). *P2ry1-KO*^*astro*^ mice also regained body weight earlier during disease progression (Supplementary Fig. S2K). Immunohistochemistry analysis revealed a loss of NeuN-positive neurons in the ventral horn of the spinal cord in control mice during the chronic disease phase, but less so in *P2ry1-KO*^*astro*^ mice, indicating a neuroprotective phenotype (Fig. 2I). Moreover, both the mean fluorescence intensity and area of GFAP-positive astrocytes were reduced in the spinal cords of *P2ry1-KO*^*astro*^ mice (Fig. 2J, Supplementary Fig. S2L), whereas no significant differences were detected in the number of infiltrating CD3⁺ T cells or Iba1⁺ microglia (Fig. 2K; Supplementary Fig. S2M-N).

Collectively, these findings identify astrocytic P2Y_1_ as an inflammation-responsive receptor that promotes ERK signalling and contributes to chronic EAE severity. Its genetic deletion in astrocytes confers neuroprotection independent of altered T cell infiltration.

### P2Y_1_-dependent astrocytic activation induces a proinflammatory phenotype

**Fig. 3:**
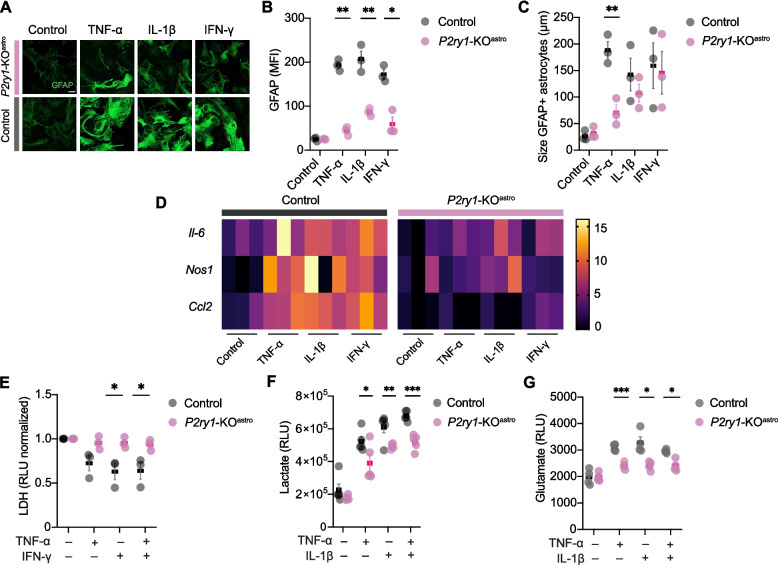
P2Y_1_-dependent astrocytic activation induces a pro-inflammatory phenotype. **A** Representative images of stimulated primary astrocytic cultures (PAC) corresponding to the quantitative data shown in (**B**) and (**C**). **B** Comparison of astrocytic activation after 8 h stimulation with respective cytokines, assessed by GFAP mean fluorescence intensity (MFI) in *P2ry1*-proficient (control) and *P2ry1*-deficient PACs (*P2ry1-KO*^*astro*^) (*n* = 3 per group). **C** Quantification of astrocytic activation after 8 h cytokine stimulation, measured as the increase in maximum cell diameter including processes corresponding to astrocytic hypertrophy in *P2ry1*-proficient (control) and *P2ry1*-deficient PACs (*P2ry1*-KO^astro^) (*n* = 3 per group). **D** Heatmap of *tbp*–normalized qPCR data (2^−ΔCT^) and quantitative analysis of inflammation- and neurotoxicity-associated genes (*IL-6, Nos1, Ccl2*) from primary astrocytic KO cultures (*P2ry1*-KO^astro^) and littermate controls after 24 h stimulation with cytokines. Colour code indicating the relative expression. **E** Relative survival of cells of the mouse neuroblastoma-derived neuronal cell line Neuro2a (N2a) pre-activated with IFN-γ (100 ng mL^–1^) and subsequently exposed to conditioned media from wild-type and *P2ry1*-deficient astrocytic cultures evaluated by LDH release assay. Astrocytes were treated with IL-1β (10 ng mL^–1^) and TNF-α (10 ng mL^–1^) for 8 h. **F** Quantification of lactate secretion by luminescence assay in primary astrocytes from wild-type and *P2ry1*-deficient mice after 8 h stimulation with IL-1β (10 ng mL^–1^) and TNF-α (10 ng mL^–1^) (*n* = 5 per group). **G** Quantification of glutamate secretion by luminescence assay in primary astrocytes from wild-type and *P2ry1*-deficient mice after 8 h stimulation with IL-1β (10 ng mL^–1^) and TNF-α (10 ng mL^–1^) (*n* = 4 per group). Data are presented as mean ± SEM (**B**, **C**, and **E**–**G**). In **B**, **C** and **E**–**G** two-way ANOVA with FDR correction was performed. Data in **D** are presented as relative gene expression values.; *P < 0.05, **P < 0.01, ***P < 0.001. Scale bar = 20 µm

We next assessed P2Y_1_-dependent astrocytic responses in vitro using PACs derived from *P2ry1*^*flx/flx*^ mice transduced with recombinant adeno-associated virus (rAAV) in which the Cre recombinase was driven by a ubiquitous CMV promoter or a Cre-negative control rAAV. Efficient *P2ry1* deletion efficiency was confirmed by RT-PCR (Supplementary Fig. S3A). Following stimulation with TNF-α, IL-1β, or IFN-γ, immunostaining for GFAP revealed that P2Y_1_-proficient astrocytes displayed significantly higher GFAP expression (Fig. 3A, B) and increased GFAP-positive area (Fig. 3A, C) compared with *P2ry1*-deficient astrocytes, indicating enhanced activation. Moreover, expression of interleukin-6 (*Il6*), nitric oxide synthase (*Nos1*), and CC-chemokine ligand 2 (*Ccl2*) evaluated by qPCR was significantly reduced in *P2ry1*-deficient PACs after cytokine stimulation (Fig. 3D).

To evaluate the impact of astrocyte-derived secretory factors on neuronal viability, we transferred supernatants from cytokine-stimulated *P2ry1*-deficient or control astrocytes onto Neuro2a neuronal cells. Neuronal viability, assessed by lactate dehydrogenase (LDH) release, was significantly reduced following exposure to supernatants from control astrocytes, but not from *P2ry1*-deficient astrocytes (Fig. 3E). No differences were observed under unstimulated conditions (Supplementary Fig. S3B). Given the importance of astrocytic metabolic support, we next quantified lactate concentrations in the culture supernatants. Stimulation with TNF-α, IFN-γ, or both increased lactate production in both groups; however, lactate levels were significantly lower in *P2ry1*-deficient PACs (Fig. 3F, Supplementary Fig. S3C). In addition, extracellular glutamate concentrations following cytokine stimulation were significantly lower in *P2ry1*-deficient astrocytes compared with control cultures (Fig. 3G).

These findings position astrocytic P2Y_1_ as an important regulator of inflammation-driven astrocyte reactivity characterized by enhanced GFAP upregulation, cytokine and metabolite release, and reduced neuronal viability.

### Neuronal P2Y_1_ signalling modulates excitotoxic and oxidative stress responses

**Fig. 4:**
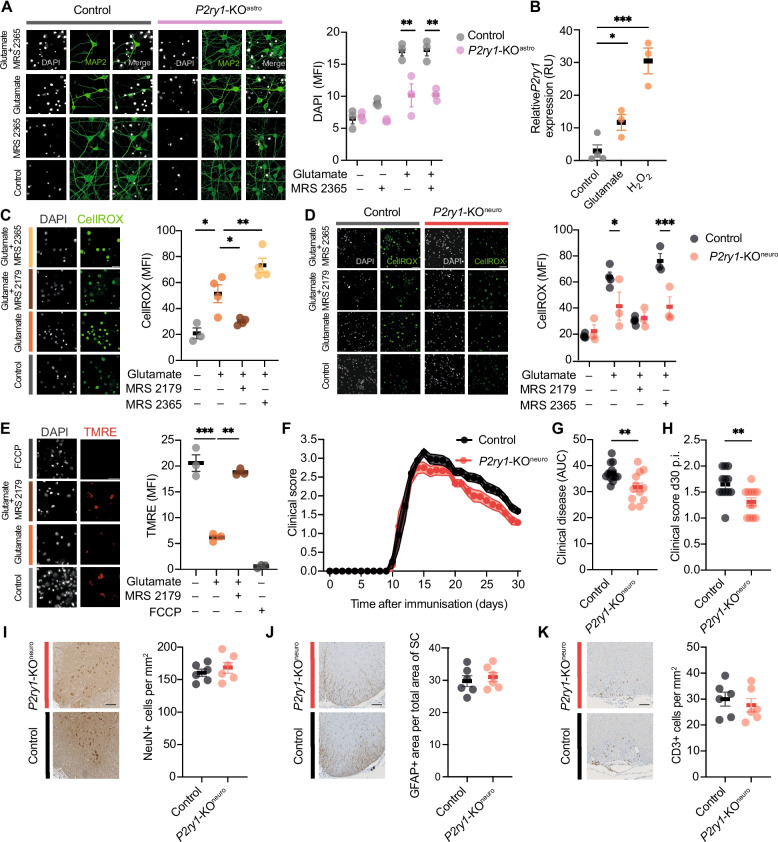
Neuronal P2Y_1_ regulates vulnerability, oxidative stress, and neuroinflammation. **A** DAPI uptake by bottom-layer primary cortical neurons in an astrocytic–neuron co-culture model with *P2ry1*-deficient astrocytes under different treatment conditions. Cells were pretreated with the P2Y_1_ agonist MRS 2365 (10 µM, 1 h) and subsequently exposed to glutamate (50 µM, 6 h) (*n* = 3 per group). Scale bars = 20 µm. **B** Relative neuronal *P2ry1* mRNA expression (relative unit = RU) following 6 h treatment with glutamate (5 µM) or H₂O₂ (10 µM), normalized to *Tbp* (*n* = 3–4). **C** Quantification of oxidative stress (CellROX mean fluorescence intensity, MFI) in primary cortical neurons. Cells were pretreated with MRS 2365 (1 µM) or MRS 2179 (10 µM) for one hour before stimulation with glutamate (5 µM) or H₂O₂ (10 µM) for an additional hour (*n* = 3–4). Scale bars = 20 µm (**D**) Quantification of oxidative stress (CellROX, MFI) in *P2ry1*-proficient (control) and *P2ry1*-deficient primary cortical neurons. Cells were pretreated with either MRS 2365 (1 µM) or MRS 2179 (10 µM) for one hour followed by stimulation with glutamate (5 µM, 1 h) (*n* = 3). Scale bars = 10 µm (**E**) Quantification of mitochondrial membrane potential using the fluorescent dye tetramethylrhodamine ethyl ester (TMRE) in primary neuronal cultures. Cells were pretreated with MRS 2179 (10 µM, 1 h) followed by glutamate stimulation (5 µM, 1 h). FCCP served as a negative control (*n* = 3). Scale bars = 20 µm. **F**–**H** **F** Disease course and **G** clinical score measured by area under the curve (AUC) during peak and chronic disease stage (15–30 days p.i.) and **H** d30 p.i. of control and *P2ry1-KO*^*neuro*^ mice subjected to EAE (control, *n* = 13; *P2ry1-KO*^*neuro*^, *n* = 12). **I**-**K** Histopathological quantification of (**I**) neuronal loss (NeuN), **J** astrocytic activation and **K** T-cell infiltration during the chronic phase, 30 days post-immunisation (all groups, *n* = 6). Scale bar = 100 µm. NeuN-positive cells were quantified as cells per mm^2^ in the ventral horn of the spinal cord (SC), whereas GFAP-positive area per total area and number of CD3-positive cells were quantified across the entire spinal cord section. In **A**, neuronal viability was assessed by DAPI uptake in live cells prior to fixation. In **C**-**E**, DAPI was added shortly before imaging for visualisation of cell nuclei for assessment of total cell number and was not used for quantitative assessment of membrane permeability or cell viability in these experiments. Data are presented as mean ± SEM (**A**–**E** and **G**–**K**). In **A**, **C** and **D**, two-way ANOVA with FDR correction was performed. Data in **B** and **E** were analysed using one-way ANOVA with FDR correction. In **F**, clinical scores were analysed using two-way repeated-measures ANOVA with FDR correction. For **G**-**K**, two-sided Mann–Whitney tests were performed.; *P < 0.05, **P < 0.01, ***P < 0.001

To further explore P2Y_1_-mediated neurotoxicity, we established co-cultures of PACs and PNCs using transwell assays. Exposure to 10 µM glutamate for 6 h increased neuronal cell death, as assessed by DAPI staining, in co-cultures containing P2Y_1_-proficient but not *P2ry1*-deficient astrocytes (Fig. 4A). Addition of the P2Y_1_ agonist MRS 2365 further increased neurotoxicity in co-cultures with P2Y_1_-proficient astrocytes but did not exacerbate glutamate-induced toxicity (Fig. 4A). To evaluate neuron-intrinsic contributions, we analysed PNCs independently. Exposure to glutamate or H_2_O_2_, significantly upregulated neuronal *P2ry1* expression (Fig. 4B). Pharmacological inhibition of P2Y_1_ using the antagonist MRS 2179 reduced cell death. However, these effects did not reach statistical significance (Supplementary Fig. S4A). Morphological analyses of the primary neuronal cultures revealed a significant reduction in rarefication of dendritic branches in neurons treated with the antagonist MRS 2179 (Supplementary Fig. S4B-C). Furthermore, expression of the immediate early gene (IEG) *c-fos* measured by qPCR was reduced following P2Y_1_ inhibition by MRS 2179 during H_2_O_2_ exposure (Supplementary Fig. S4D).

Because oxidative stress and mitochondrial dysfunction are central features of neuroinflammation, we next assessed reactive oxygen species (ROS) production using the CellROX assay. Glutamate exposure for one hour markedly increased ROS levels, which were attenuated by the P2Y_1_ antagonist MRS 2179 and potentiated by the P2Y_1_ agonist MRS 2365 (Fig. 4C). These results were replicated in the mouse hippocampal cell line mHippoE-14 (Supplementary Fig. S4E). Consistently, ROS production was significantly lower in PNCs derived from *P2ry1*^*flx/flx*^ mice transduced with AAV-CMV-Cre following glutamate stimulation (Fig. 4D). This reduction was accompanied by decreased loss of mitochondrial membrane potential (ΔΨm), an effect that was further mitigated by pharmacological P2Y_1_ inhibition (Fig. 4E).

Together, these findings demonstrate that P2Y_1_ receptor inhibition exerts protective effects in both astrocytes and neurons. Consistent with this, neuron-specific deletion of *P2ry1* (*P2ry1-KO*^*neuro*^) significantly reduced EAE severity, as reflected by lower cumulative and chronic-phase scores (Fig. 4F-H, Supplementary Fig. S4F). However, the clinical benefit was less pronounced than that observed following astrocyte-specific *P2ry1* deletion, and no significant histological neuroprotection was detected at 30 days post-immunisation, as assessed by NeuN, GFAP, CD3 or Iba1 staining (Fig. 4I-K, Supplementary Fig. S4G-H). Overall, our results identify astrocytes as the primary mediators of P2Y_1_ -driven neurodegeneration during CNS inflammation.

## Discussion

This study identifies astrocytic P2Y_1_ receptor signalling as a key driver of neuroinflammation-induced neurodegeneration in EAE. We show that extracellular ATP levels increase during the acute disease phase, coinciding with upregulation of *P2ry1* in astrocytes. Genetic or pharmacological inhibition of astrocytic P2Y_1_ reduced disease severity, attenuated astrocytosis, and preserved neuronal integrity, whereas neuron-specific *P2ry1* deletion conferred only modest clinical benefit without structural protection. These findings underscore astrocyte-specific P2Y_1_ signalling as a critical effector of inflammation-driven neuronal injury.

Our discoveries extend previous observations that reactive astrocytes undergo extensive purinergic remodelling during neuroinflammation and neurodegeneration. *P2ry1* upregulation has been shown to enhance aberrant astrocytic Ca^2^⁺ signalling and neuronal hyperexcitability in models of Alzheimer’s disease and epilepsy [39, 40], while other studies under acute oxidative stress conditions proposed a protective astrocytic P2Y_1_ –IL-6 axis [41]. Our data reconcile these apparently divergent observations by demonstrating that, in the context of chronic autoimmune neuroinflammation, sustained P2Y_1_ activation promotes a deleterious reactive astrocyte phenotype characterized by increased cytokine and chemokine expression, impaired metabolic support, and enhanced neurotoxicity.

Mechanistically, these effects are likely mediated through the canonical Gαq–PLCβ–Ca^2^⁺ signalling pathway downstream of P2Y_1_. Binding of ATP or ADP induces intracellular Ca^2^⁺ release and protein kinase C activation, which converge on NF-κB-dependent transcriptional programs that drive the expression of IL-6, iNOS, and CCL2. This pathway has been recognized as a central link between purinergic signalling and inflammatory gene induction in astrocytes and microglia [42]. In our model, these transcriptional changes coincided with altered astrocyte metabolism, reflected by reduced lactate release, suggesting that P2Y_1_ activation not only promotes inflammation but also impairs astrocyte-neuron metabolic coupling. Similar metabolic reprogramming toward an energy-deficient, pro-oxidant phenotype has been described in reactive astrocytes during chronic neuroinflammation [15, 43, 44]. Collectively, these data suggest that P2Y_1_ signalling destabilizes astrocyte–neuron homeostasis, transforming astrocytes from supportive cells to neurotoxic effectors.

This astrocytic pathway likely operates within a broader purinergic feed-forward loop at the glia–neuron interface [45–47]. Elevated extracellular ATP, released from stressed neurons or activated glia, stimulates P2Y_1_ on neighbouring astrocytes, inducing further ATP release via connexin and pannexin channels [48–50]. This signalling propagates intercellular Ca^2^⁺ waves and excitatory gliotransmission [39, 51]. Under inflammatory conditions, such ATP-driven signalling may sustain a self-perpetuating cycle of astrocyte activation and neuronal hyperexcitability, consistent with our observation that supernatants from P2Y_1_ -proficient astrocytes increased neuronal vulnerability to glutamate-induced excitotoxicity. Although neuronal P2Y_1_ activation enhanced ROS production and mitochondrial depolarization in vitro, our in vivo findings indicate that these neuronal effects are mostly secondary to astrocyte-mediated toxicity, underscoring the dominant role of glial P2Y_1_ in driving neurodegeneration.

### Limitations of the study

Several limitations of this study should be acknowledged. Pharmacological inhibition of P2Y_1_ by intranasal administration of MRS 2179 resulted in only modest effects on disease severity. Interpretation of this experiment is limited by the relatively small cohort size and by the limited availability of the compound in the relevant target compartment, due to restricted blood–brain barrier penetration and a short serum half-life. These factors may have compromised the statistical power to detect moderate treatment effects.

For this reason, the study subsequently focused on conditional cell type-specific *P2ry1* knockout models to more directly address the contribution of CNS-resident P2Y_1_ signalling to neuroinflammatory pathology.

These data then demonstrated a prominent role of astrocytic P2Y_1_ during inflammation-induced neurodegeneration. However, a direct mechanistic link between astrocytic P2Y_1_ signalling and astrocyte-mediated neuronal damage was not formally established, and a more indirect role through modulation of the inflammatory milieu remains possible. While CD3⁺ T-cell infiltration, astrocytic inflammatory gene expression, and Iba1 immunoreactivity were analysed, a more detailed characterisation of CNS myeloid responses could be instructive. Notably, Iba1 immunoreactivity was not significantly altered in diseased astrocyte-specific *P2ry1* knockout mice, suggesting that the observed neuroprotective effects are not simply attributable to a broad suppression of myeloid activation. However, Iba1 staining alone does not resolve potential changes in microglial functional states, inflammatory polarisation, or phagocytic activity.

In addition, although astrocyte-specific *P2ry1* deletion confirms a central glial mechanism, potential contributions from other CNS cell types, such as oligodendrocytes, were not examined. Moreover, astrocytes constitute a highly heterogeneous population [15, 52], and future studies should determine how distinct astrocytic subtypes contribute to P2Y_1_-dependent pathomechanisms. Further mechanistic studies are required to define the subcellular P2Y_1_-dependent mechanisms in both neurons and astrocytes. Although pharmacological inhibition of P2Y_1_ attenuated glutamate-induced mitochondrial depolarisation, mitochondrial membrane potential was not directly assessed in *P2ry1*-deficient neurons.

## Conclusions

In conclusion, our study identifies astrocytic P2Y_1_ receptor signalling as an important mediator of glia-neuron communication during neuroinflammation and a key regulator of astrocyte-driven inflammatory and metabolic dysfunction. These findings position P2Y_1_ as a potential therapeutic target for modulating neurotoxic glial responses and limiting tissue injury in autoimmune diseases such as MS.

## Supplementary Information

Supplementary Material 1.

## Data Availability

All data supporting the conclusions of this study are included in the main text and/or the Supplementary Information.
