# Supplementary material for: Neuroglial P2Y1 receptor signalling differentially contributes to inflammatory neurodegeneration
Source: J Neuroinflammation. 2026 Jun 13;23:199. doi: 10.1186/s12974-026-03904-1 (PMC13263940; doi:10.1186/s12974-026-03904-1)
Supplement: Supplementary file 1 — Supplementary Material 1. [file 12974_2026_3904_MOESM1_ESM.pdf]

SUPPLEMENTARY INFORMATION

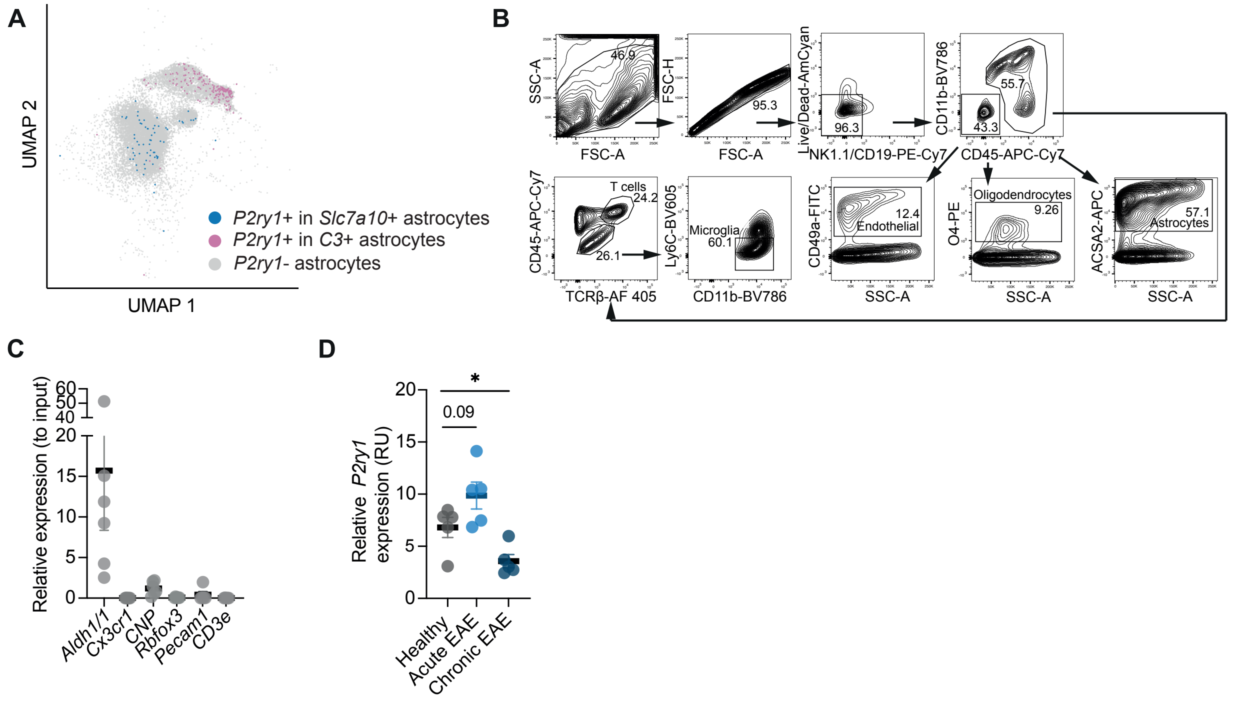

Supplementary Fig. S1. Expression of P2RY1 in astrocytes during neuroinflammation.

(A) UMAP visualisation of spinal cord single-cell RNA-sequencing data (GSE281176, n = 4 animals) comprising 93,793 nuclei from healthy C57BL/6J mouse spinal cord highlighting *P2ry1*-positive astrocyte populations within the astrocyte clusters. **(B)** Gating strategy for astrocyte isolation from brain and spinal cord in C57BL/6J mice. **(C)** Validation of sorted astrocytes by RT-PCR, confirming astrocyte enrichment (*Aldh1l1*) and depletion of microglia (*Cx3cr1*), oligodendrocytes (*Cnp*), neurons (*Rbfox3*), endothelial cells (*Pecam1*), and T cells (*CD3ε*) compared with the input population (n = 6 per group). **(D)** Relative *P2RY1* expression measured by RT-PCR (arbitrary unit, AU) in sorted astrocytes from healthy controls, acute EAE, and chronic EAE (n = 5 per group). Data are shown as the mean ± s.e.m. (C and D). In B, a representative gating strategy is shown. In C and D, one-way ANOVA with FDR correction was performed.; *P < 0.05.

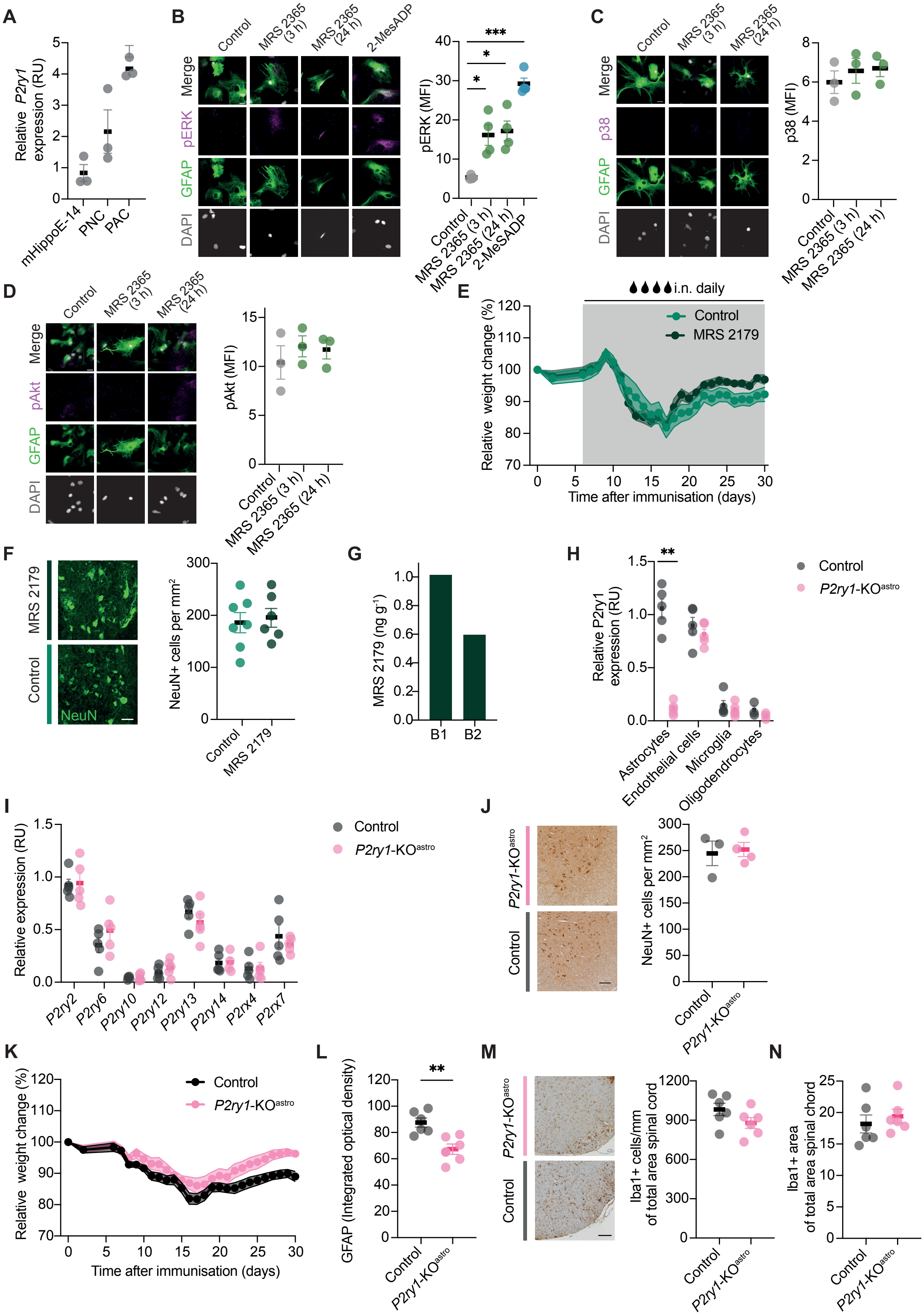
A
Relative P2ry1 expression (RU)
mHippoE-14
PNC
PAC
B
Control
MRS 2365 (3 h)
MRS 2365 (24 h)
2-MesADP
Merge
pERK
GFAP
DAPI
pERK (MFI)
***
*
*
C
Control
MRS 2365 (3 h)
MRS 2365 (24 h)
Merge
p38
GFAP
DAPI
p38 (MFI)
D
Control
MRS 2365 (3 h)
MRS 2365 (24 h)
Merge
pAkt
GFAP
DAPI
pAkt (MFI)
E
i.n. daily
Control
MRS 2179
Relative weight change (%)
Time after immunisation (days)
F
MRS 2179
Control
NeuN
NeuN+ cells per mm²
Control
MRS 2179
G
MRS 2179 (ng g⁻¹)
B1
B2
H
**
Control
P2ry1-KOastro
Relative P2ry1 expression (RU)
Astrocytes
Endothelial cells
Microglia
Oligodendrocytes
I
Relative expression (RU)
Control
P2ry1-KOastro
P2ry2
P2ry6
P2ry10
P2ry12
P2ry13
P2ry14
P2rx4
P2rx7
J
P2ry1-KOastro
Control
NeuN+ cells per mm²
K
Control
P2ry1-KOastro
Relative weight change (%)
Time after immunisation (days)
L
**
GFAP (Integrated optical density)
Control
P2ry1-KOastro
M
P2ry1-KOastro
Control
Iba1+ cells/mm of total area spinal cord
N
Iba1+ area of total area spinal chord

Supplementary Fig. S2. Analyses of $P2Y_1$ downstream signalling and in vivo responses.

(A) Comparison of relative *P2ry1* mRNA expression (relative unit, RU) in mHippoE-14 cell line, primary astrocytic cultures (PACs), and primary neuronal cultures (PNCs) (n = 3 per group). **(B-D)** Representative images and quantification of mean fluorescence intensity (MFI) of (B) phospho-ERK1/2, (C) p38 MAPK and (D) phospho-AKT in primary astrocytes following stimulation with the $P2Y_1$-agonist MRS 2365 (1 µM) for 3 hours or 24 hours. Phospho-ERK1/2 MFI was also assessed following stimulation with the non-selective $P2Y_1$ agonist 2-methylthioadenosine diphosphate (2-MeSADP) for 24 hours. (pERK MFI, n = 3-4; p38 MAPK MFI, n = 3; pAKT MFI, n = 3). Scale bar = 20 µm. **(E)** Time course of relative body weight change during EAE in mice treated daily with PBS (control) or MRS 2179 (10 mg kg^{-1}, intranasal; n = 7 per group). **(F)** Representative images and histopathological quantification of neuronal loss (NeuN) (MRS 2179 group, n = 6; control group, n = 7). Scale bar = 100 µm. **(G)** Concentration of MRS 2179 (ng g^{-1}) brain tissue (sample 1 = B1, sample 2 = B2) of C57BL/6J mice treated intranasally with MRS 2179 (10 mg kg^{-1}) three hours prior to dissection. Measurements were performed by high-performance liquid chromatography coupled to mass spectrometry (HPLC-MS). **(H)** Relative *P2ry1* mRNA expression (RU) in astrocytes, endothelial cells, microglia, and oligodendrocytes isolated from control and astrocyte-specific *P2ry1* knockout mice (*P2ry1*-KO^{astro}) measured by RT-PCR (n = 5 per group). **(I)** Relative mRNA expression (RU) of purinergic receptors in astrocytes isolated from control and astrocyte-specific *P2ry1* knockout mice (*P2ry1*-KO^{astro}) (n = 5 per group). **(J)** Representative images and histopathological quantification of $NeuN^+$ cell density in the ventral horn spinal cord area from healthy control and astrocyte-specific *P2ry1* knockout mice (P2ry1-KO^{astro}) (n = 3-4 per group). Scale bar = 100 µm. **(K)** Time course of relative body weight change during EAE in astrocyte-specific *P2ry1* conditional knockout mice (*P2ry1-KO^{astro}*, n = 9) and littermate controls (control, n =13). **(L)** Histopathological quantification of astrocytic activation during chronic disease phase in the spinal cord (SC), measured by integrated optical density of GFAP in the SC (n = 6 per group). The corresponding representative is shown in Fig. 2. **(M-N)** Representative images and histopathological quantification of **(M)** $Iba1^+$ cell density and **(N)** $Iba1^+$ area in the total spinal cord area from control and astrocyte-specific *P2ry1* knockout mice (*P2ry1*-KO^{astro}) (n = 6 per group). Scale bar = 100 µm. In B-D, DAPI was added post fixation for visualisation of cell nuclei and was not used for quantitative assessment of membrane permeability or cell viability in these experiments. NeuN-positive cells were quantified as cells per mm² in the ventral horn of the spinal cord (SC), whereas GFAP-positive area per total area and number of Iba1-positive cells as well as Iba1-positive area per total area were quantified across the entire spinal cord section. Data are shown as the mean ± s.e.m. (A–D, F-N). In A–D, one-way ANOVA with FDR correction was performed. In E and K, relative weight change was analysed using two-way repeated-measures ANOVA with FDR correction. For F, H, I, J, L, M and N, two-sided Mann–Whitney tests were performed.; *$P < 0.05$, **$P < 0.01$, ***$P < 0.001$.

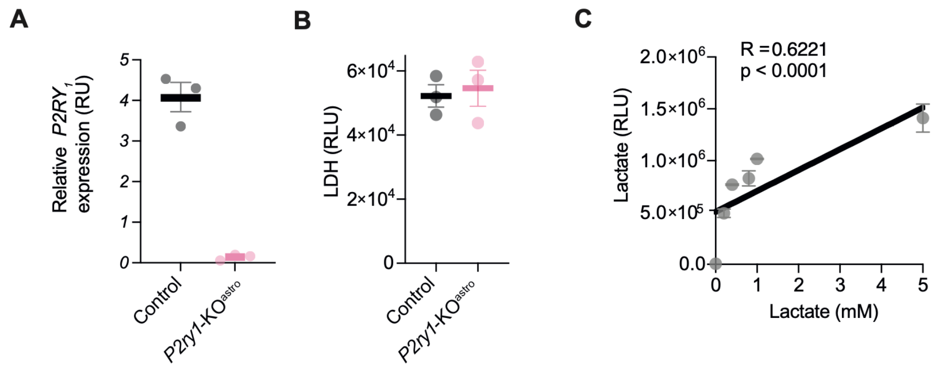

Supplementary Fig. S3. Validation of *P2ry1*-deficient astrocytic cultures and downstream experiments

(A) Relative *P2ry1* mRNA expression (relative unit, RU) in primary astrocytic cultures derived from *P2ry1*$^{flx/flx}$ mice measured by RT-qPCR. Gene knockout was induced by transduction with a Cre-expressing rAAV under control of the CMV promoter (n = 3 per group). **(B)** Baseline lactate dehydrogenase (LDH) levels measured by luminescence (relative light units, RLU) of N2a cells used for cytotoxicity assessment following supernatant exchange (n = 3 per group). **(C)** Quantification of luminescence at defined lactate concentrations using a luminescence-based assay. A linear regression model was fitted, and correlation significance was statistically evaluated (n = 3 per group). Correlation analysis was performed using Pearson's correlation coefficient

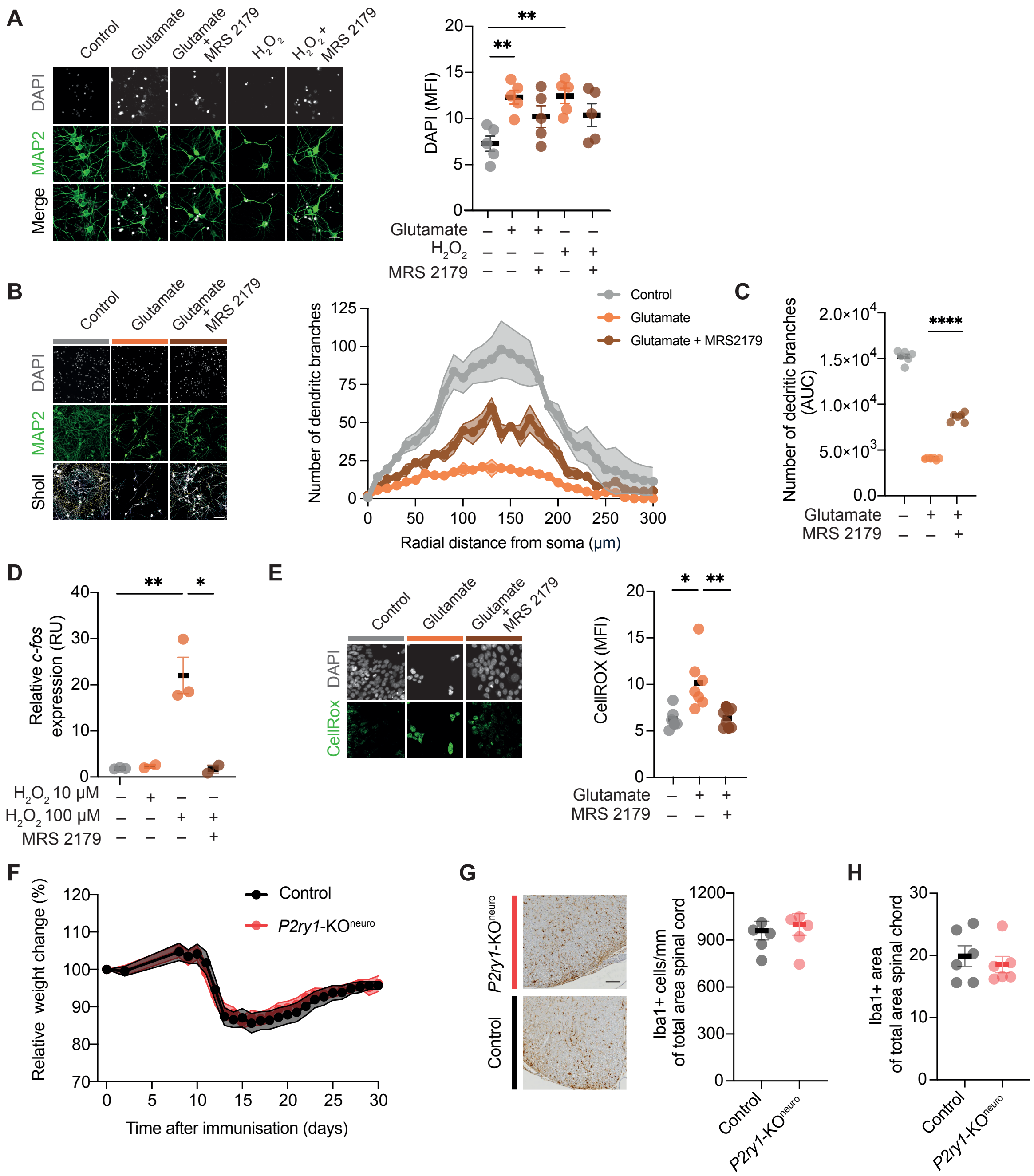

Supplementary Fig. S4. Characterisation of neuronal responses to oxidative and excitotoxic stress

(A) DAPI uptake by primary cortical neurons under different treatment conditions. Cells were pretreated with the $P2Y_1$ antagonist MRS 2179 (10 µM, 1 hour) and subsequently exposed to glutamate (5 µM, 6 hours) or H_2O_2 (10 µM, 6 hours) (all groups, *n* = 5). Scale bars = 20 µm. **(B)** Quantification of dendritic branching by using Sholl analysis at increasing distances from the soma. Cells were pretreated with the P2Y1 antagonist MRS 2179 (10 µM, 1 hour) and subsequently exposed to glutamate (5 µM, 6 hours) (all groups, *n* = 6). **(C)** Cumulative quantification of the total dendritic branching, represented as area under the curve (AUC). Cells were pretreated with the P2Y1 antagonist MRS 2179 (10 µM, 1 hour) and subsequently exposed to glutamate (5 µM, 6 hours) (all groups, *n* = 6). **(D)** Relative mRNA expression (relative unit = RU) of immediate early gene *c-fos* in primary neuronal cultures. Cells were treated with H_2O_2 (10 µM or 100 µM) or medium control for 6 hours. Where indicated, cells were pretreated with the $P2Y_1$ antagonist (10 µM) for 1 hour (*n* = 3 per group). **(E)** Quantification of oxidative stress (CellROX; mean fluorescence intensity, MFI) in the neuronal mHippoE-14 cell line. Cells were pretreated with MRS 2179 (10 µM) for one hour prior stimulation with glutamate (150 µM) for an additional hour (*n* = 6-8 per group). **(F)** Time course of relative weight change during EAE in neuron-specific *P2ry1*

knockout mice (*P2ry1-KO*^{neuro}, *n* = 12) and littermate controls (control, *n* =13). **(G-H)** Representative images and histopathological quantification of **(G)** Iba1^{+} cell density and **(H)** Iba1^{+} area in the total spinal cord area from control and neuron-specific *P2ry1* knockout mice (*P2ry1*-KO^{neuro}, *n* = 6 per group). Scale bar = 100 µm. In A, Neuronal viability was assessed by DAPI uptake in live cells prior to fixation. In B and E, DAPI was added shortly before imaging for visualisation of cell nuclei and was not used for quantitative assessment of membrane permeability or cell viability in these experiments. Data are presented as mean ± SEM (A-E). In A–E and G-H, data were analysed using one-way ANOVA with FDR correction. In F, relative weight change was analysed using two-way repeated-measures ANOVA with FDR correction.; *P < 0.05, **P < 0.01, ***P < 0.001.

Supplementary Table 1: List of antibodies used in the study.

Antigen	Supplier	Prod. no.	RRID	Species/ Isotype	Concent ration
ACSA-2 - APC	Miltenyi	130-117-535	AB_2727421	Rat	1:100
CD3ε	Abcam	ab16669	AB_443425	Rabbit	1:200
CD11b – BV786	BioLegend	101243	AB_2740514	Rat	1:200
CD19 – PE-Cy7	BioLegend	115506	AB_314246	Rat	1:200
CD45 – APC-Cy7	BioLegend	103116	AB_2868859	Rat	1.200
CD49a – Vio Bright FITC	Miltenyi	130-125-102	AB_2658435	Human	1:100
GFAP	Abcam	ab4674	AB_304558	Chicken	1:500
HuC/HuD	Invitrogen	A-21271	AB_221448	Mouse	1:500
Iba1	Fujifilm Wako	019–19741	AB_839504	Rabbit	1:1000
Ig chicken Alexa Fluor 488	Jackson Immuno	703-545-155	AB_2340375	Donkey	1:500
Ig chicken Alexa Fluor 647	Jackson Immuno	703-606-155	AB_2340380	Donkey	1:500
Ig mouse Alexa Fluor 647	Jackson Immuno	715-605-151	AB_2340863	Donkey	1:500
Ig mouse Alexa Fluor 647	Abcam	ab175658	AB_2890037	Donkey	1:500
Ig rabbit Alexa Fluor 488	Abcam	ab170073	AB_2636877	Donkey	1:500
Ig rabbit Alexa Fluor 555	Abcam	ab150074	AB_2636997	Donkey	1:500
Ig rabbit Alexa Fluor 647	Abcam	ab150105	AB_2732856	Donkey	1:500
Ly-6C – BV605	BD	563011	AB_2737949	Rat	1:200
MAP2	Abcam	ab5392	AB_2138153	Chicken	1:1000
NeuN	Merck Millipore	MAB377	AB_2298772	Chicken	1:200
NK1.1 – PE-Cy7	BioLegend	108714	AB_389364	Mouse	1:300
O4 - PE	Miltenyi	130-117-823	AB_2751913	Human	1:200
Phospho-p38 MAPK	Cell Signaling Technology	9211	AB_331641	Rabbit	1:600
Phospho-Akt	Cell Signaling Technology	9271	AB_329825	Rabbit	1:100
Phospho-ERK1/ERK2	Invitrogen	36-8800	AB_2533283	Rabbit	1:500
TCR-β – BV421	BioLegend	109230	AB_2562562	Armenian Hamster	1:200

Supplementary Table 2: List of all gene expression assays used in the study.

Gene expression assay	Supplier	Catalogue number
Aldh1l1	Thermo Fisher Scientific	Mm03048957_m1
CD3ε	Thermo Fisher Scientific	Mm01179194_m1
CCl2	Thermo Fisher Scientific	Mm00441242_m1
CNP	Thermo Fisher Scientific	Mm01306641_m1
Cx3cr1	Thermo Fisher Scientific	Mm00438354_m1
Fos	Thermo Fisher Scientific	Mm00487425_m1
IL-6	Thermo Fisher Scientific	Mm00446190_m1
Nos1	Thermo Fisher Scientific	Mm00440502_m1
Pecam1	Thermo Fisher Scientific	Mm01242576_m1
P2rx4	Thermo Fisher Scientific	Mm00501787_m1
P2rx7	Thermo Fisher Scientific	Mm01199500_m1
P2ry1	Thermo Fisher Scientific	Mm02619947_m1
P2ry2	Thermo Fisher Scientific	Mm02619978_s1
P2ry6	Thermo Fisher Scientific	Mm01275476_m1
P2ry10	Thermo Fisher Scientific	Mm02620706_s1
P2ry12	Thermo Fisher Scientific	Mm00446026_m1
P2ry13	Thermo Fisher Scientific	Mm00546978_m1
P2ry14	Thermo Fisher Scientific	Mm01289602_m1
Rbfox3	Thermo Fisher Scientific	Mm01248771_m1
Tbp	Thermo Fisher Scientific	Mm01277042_m1
